# OrthoGuide: A Database for Rooting Inference of Orthologous Genes

**DOI:** 10.1093/gbe/evag119

**Published:** 2026-06-19

**Authors:** João V F Cavalcante, Gleison M de Azevedo, Danilo O Imparato, Diego Marques-Coelho, Mauro A A Castro, Rodrigo J S Dalmolin

**Affiliations:** Bioinformatics Multidisciplinary Environment, UFRN, R. do Horto - Lagoa Nova, Natal, RN 59076-550, Brazil; Bioinformatics Multidisciplinary Environment, UFRN, R. do Horto - Lagoa Nova, Natal, RN 59076-550, Brazil; Bioinformatics Multidisciplinary Environment, UFRN, R. do Horto - Lagoa Nova, Natal, RN 59076-550, Brazil; Bioinformatics Multidisciplinary Environment, UFRN, R. do Horto - Lagoa Nova, Natal, RN 59076-550, Brazil; Bioinformatics and Systems Biology Laboratory, Federal University of Paraná, Curitiba, PR 81520-260, Brazil; Bioinformatics Multidisciplinary Environment, UFRN, R. do Horto - Lagoa Nova, Natal, RN 59076-550, Brazil; Department of Biochemistry, UFRN, Av. Sen. Salgado Filho, 3000 - Lagoa Nova, Natal, RN 59064-741, Brazil

**Keywords:** evolutionary rooting, systems evolution, orthology, database, web application

## Abstract

Orthology provides a powerful framework for investigating the evolutionary history of biological systems, as genes within the same orthologous group typically share a common ancestry. By tracing their distribution across species, it is possible to infer the evolutionary origin of genes and reconstruct the stepwise assembly of molecular pathways and regulatory networks. However, performing such analyses at scale often requires specialized computational tools and expertise, limiting their accessibility to a broader community. Here, we introduce OrthoGuide, a database and web application that provides precomputed evolutionary rooting information for orthologous groups across 360 eukaryotic species. The platform enables users to query gene sets and rapidly explore their evolutionary origins through an intuitive interface, without the need for local computational workflows. In addition to tabular outputs, OrthoGuide offers interactive visualizations that facilitate the interpretation of evolutionary patterns and the identification of key events in the emergence of biological systems. By removing technical barriers and standardizing large-scale evolutionary inferences, OrthoGuide enables researchers to translate gene lists into biologically meaningful hypotheses. This resource democratizes access to orthology-based evolutionary analyses and supports the investigation of system-level evolutionary processes across a wide range of organisms. The web application and the database are hosted at https://dalmolingroup.imd.ufrn.br/orthoguide/.

Significance Understanding the evolutionary origin of genes is fundamental for systems biology but is often obstructed by computationally complex bioinformatic workflows. This creates a bottleneck for many researchers. OrthoGuide directly addresses this challenge by providing a public, precomputed database of evolutionary rooting data for all genes across 360 eukaryotic organisms. Our web application eliminates the need for user-side computation. OrthoGuide delivers instant results as well as interactive visualizations. This resource democratizes access to evolutionary analyses based on orthology information, enabling a broader scientific community to rapidly translate simple gene lists into insights about the assembly of biological systems.

## Introduction

Orthologous groups (OGs), which comprise genes descended from a single common ancestor, serve as a proxy for investigating the evolutionary history of complex biological systems. By tracing their distribution across a species tree, researchers can infer the evolutionary root, or point of origin, for each component of a system, such as a metabolic pathway or a particular trait. This enables a systems-level perspective that moves beyond the analysis of single genes to examine how functional networks were assembled over evolutionary time. In this context, the “root clade” corresponds to the ancestral node that best reconciles the observed phyletic distribution of an OG under a vertical inheritance model. When multiple genes from a pathway or molecular network are collectively mapped onto a phylogeny, their root placements provide a framework to reconstruct the evolutionary scenarios underlying the emergence and stepwise assembly of the system. This perspective shifts the emphasis away from estimating the absolute age of individual genes toward understanding how functionally integrated molecular systems were progressively established during evolution.

This methodology has yielded a variety of tools (Campos et al. 2024; Middlebrook et al. 2024) and significant insights into the evolution of critical cellular functions. In this context, orthologous gene information has been used to unravel the evolution of neurotransmitter and apoptosis systems (Castro et al. 2008; Viscardi et al. 2021), to construct coevolutionary gene networks in yeast species (Steenwyk et al. 2022), to describe the evolution of peroxidases in plants (Oliveira et al. 2019) and to identify key drivers of multicellularity in animal evolution (Li et al. 2025).

Established probabilistic frameworks, such as GLOOME (Cohen et al. 2010) and EREM (Carmel et al. 2010), provided robust approaches for reconstructing ancestral states and inferring gene gain and loss events across phylogenies. These methods are designed to model the dynamics of character transitions over the entire tree, rather than to directly identify the evolutionary root of OGs. Earlier approaches, such as the PARS algorithm (Mirkin et al. 2003), reconstruct parsimonious evolutionary scenarios for sets of orthologs based on their phyletic patterns in a given species tree. However, such methods are not well suited for high-throughput analyses involving large numbers of orthologous groups and species.

In this context, the Bridge algorithm (Campos et al. 2024) was developed to enable scalable inference of evolutionary roots from orthology data. By systematically evaluating the distribution of OGs across a phylogenetic tree, Bridge provides a practical framework for high-throughput rooting inference, making it particularly suitable for large-scale, systems-level evolutionary analyses. The algorithm infers a gene’s evolutionary root by systematically evaluating its phyletic pattern—the presence or absence of its orthologs—across a given species tree. A key advantage of this method is that it does not require a predefined outgroup, making it particularly well-suited for deep evolutionary analyses where a suitable outgroup may be distant or ambiguous. The Bridge algorithm operates by iteratively testing each ancestral node in the phylogenetic tree as a potential point of origin, calculating a consistency score that measures how well that root explains the observed distribution of the OG through vertical descent and subsequent gene loss. The Bridge algorithm has been made available through an R package, GeneBridge (https://github.com/sysbiolab/GeneBridge). The algorithm’s capacity for high-throughput analysis was recently demonstrated in a large-scale study of over 36,000 microprotein families across 5,668 Enterobacteriaceae genomes, where it was utilized to pinpoint the points of origin for evolutionarily young, de novo-originated genes (Fesenko et al. 2025).

Despite the power of algorithms like Bridge, their application has largely been confined to researchers with bioinformatic expertise, often requiring the use of command-line tools and package installations. This creates a technical barrier that limits the broader scientific community from leveraging these insights. OrthoGuide addresses this gap by providing a comprehensive, precomputed database of rooting inferences generated by the Bridge algorithm, along with an intuitive accompanying web application to access it.

## Results and Discussion

OrthoGuide is architected as a static web application that queries a SQLite database directly in the user’s browser via WebAssembly. This approach was chosen to significantly enhance performance, portability, and scalability. By executing all data operations on the client-side, the application delivers query results after an initial database load. This self-contained model also empowers other developers and research groups to easily extend or self-host their own instances of OrthoGuide.

To ensure the database itself is reproducible and extensible, it is constructed via a containerized Nextflow pipeline. This workflow automation tool guarantees that the entire data generation process is standardized, version-controlled and modifiable. Additionally, OrthoGuide’s interactive visualizations, while simple, are fully exportable as publication-ready Scalable Vector Graphics (SVG) files, allowing for lossless resizing and easy integration into academic manuscripts.

To evaluate OrthoGuide assessing the evolutionary history of a specific biological system, we analyzed the human Taste Transduction pathway from the KEGG database (hsa:map04742) (Kanehisa et al. 2025). In this context, the identified “root clade” represents the Last Common Ancestor (LCA) shared between the reference species (Homo sapiens) and the named clade, identifying the divergence point where the OG originated. This pathway, comprising 86 genes in humans, represents an excellent test case because it is a functional mosaic, containing both ancient, broadly conserved genes and more recently evolved, lineage-specific receptor families. The complete table of results can be acquired from Supplementary Table 1.

**Fig. 1.**
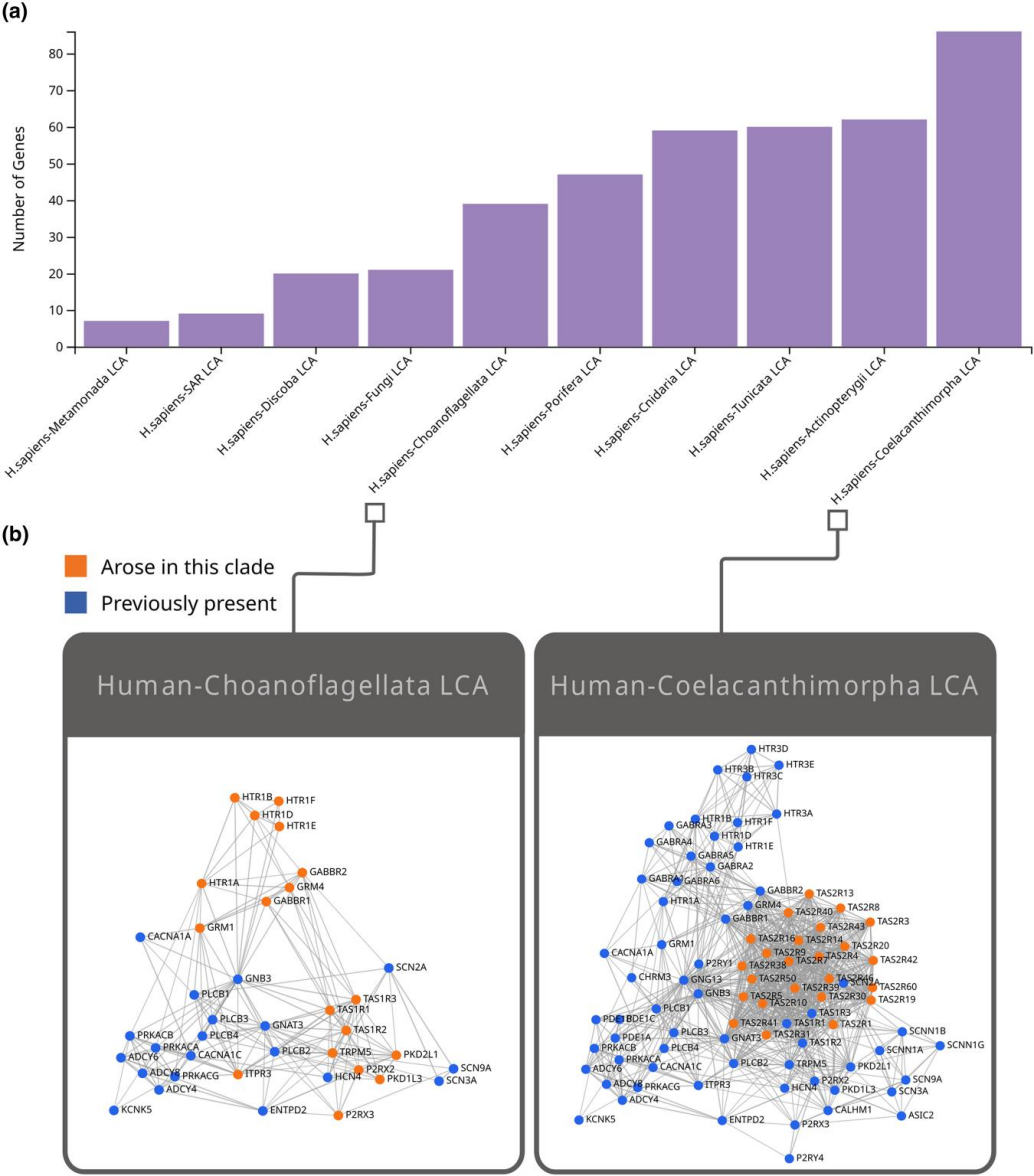
Visualizations from OrthoGuide for genes from the Taste transduction pathway. a) The cumulative number of rooted genes at each ancestral point in the species tree for this set. b) Protein-protein interaction networks considering the cumulative presence of rooted nodes from each root clade. Nodes rooted at each point are shown in orange while nodes rooted in earlier clades are shown in blue.

The analysis revealed a stepwise assembly of the taste transduction pathway, characterized by several distinct periods of gene emergence (Fig. 1). The most ancient components of the system, comprising 22 genes, were rooted in the ancestors shared between humans and clades such as Metamonada, SAR, and Discoba. These early additions represent the fundamental signaling toolkit of the cell, including core secondary messenger enzymes like adenylate cyclases (ADCY4/6/8) and phospholipase C (PLCB1-4), as well as essential G-protein subunits (GNAT3, GNB3). Their early presence suggests that the intracellular machinery required to process chemosensory signals predates the evolution of specialized taste receptor families.

Following this foundational period, a major expansion event occurred at the LCA of Choanoflagellata and humans, where 18 genes were rooted. This cohort includes the birth of umami and sweet taste receptors (TAS1R1-3) and metabolic glutamate receptors (GRM1/4), alongside critical effector channels such as TRPM5 and ITPR3. This ancient origin is biologically meaningful, as choanoflagellates are the closest known unicellular relatives of animals. These components likely formed a molecular repertoire for environmental chemosensation, consistent with known signaling capabilities in this clade (Ros-Rocher and Brunet 2023).

Further diversification of the pathway took place during the transition to multicellularity and early animal evolution. Specifically, 17 genes were rooted to the ancestors shared with Porifera and Cnidaria. This phase saw the integration of ionotropic receptors, including a large cohort of GABA receptors (GABRA1-6) and serotonin receptors (HTR3A-E), as well as epithelial sodium channel subunits (SCNN1A/B/G). These additions suggest a period of increasing complexity in how environmental signals were translated into electrical responses within emerging nervous systems.

A final significant expansion event occurred later, with 24 genes emerging in the LCA of Coelacanthimorpha and humans. This diversification is dominated by the evolution of bitter taste perception, mediated by the Taste Receptor Type 2 (T2R) family. The coelacanth is a critical node in vertebrate evolution and is noted for being the first fish lineage to possess an extensive repertoire of bitter taste receptors, likely as an adaptation to detect toxins in a more complex diet (Behrens et al. 2021; Picone et al. 2014). This evolutionary finding is also clearly visualized in our results as the emergence of a highly connected module of proteins belonging to the T2R family.

In summary, this evaluation demonstrates OrthoGuide’s ability to uncover a broader evolutionary narrative of a biological system. The results not only pinpoint key evolutionary events but also place them in their proper biological context.

## Conclusions

OrthoGuide provides a systems biology-based platform for the evolutionary analysis of genes and biological pathways, designed to identify the evolutionary origins of functionally associated genes. The core advantage of OrthoGuide over existing analytical workflows is its accessibility; it provides precomputed rooting information generated by the Bridge algorithm for 360 species, which infers the evolutionary age of a gene based on the phyletic distribution of its OG. OrthoGuide eliminates technical obstacles by presenting the results in an intuitive, query-based web application, enhanced by interactive visualizations that allow users to explore the stepwise assembly of a system’s components over time.

Future iterations of OrthoGuide will focus on expanding its taxonomic and analytical breadth. This includes the integration of diverse orthology sources, such as the OMA database (Altenhoff et al. 2018), to allow for cross-database validation and broader gene coverage. Additionally, while the current release is centered on eukaryotes, the potential for expanding into prokaryotic systems is supported by the successful application of the Bridge algorithm to large-scale bacterial datasets. Such expansions would enable the platform to support a more comprehensive range of evolutionary questions across the tree of life.

It is important, however, to acknowledge the limitations inherent to this precomputed approach. The accuracy of OrthoGuide’s inferences is dependent on the quality and comprehensiveness of the curated orthology database it is built upon. Any inaccuracies in the underlying orthology assignments will naturally propagate into our results. The orthology community is actively addressing such challenges through initiatives like the Quest for Orthologs consortium (Altenhoff et al. 2024), which focuses on standardizing benchmarking procedures and improving data interoperability between orthology resources. By grounding OrthoGuide in curated community datasets, we aim to provide an accessible entry point for evolutionary analysis that benefits from these ongoing improvements in orthology assignment accuracy. Furthermore, because the database is static, users cannot infer new evolutionary scenarios using custom phylogenetic trees or for species not yet included in our dataset. This trade-off is by design, as it enables the platform’s primary strength: delivering instantaneous, resource-free analysis for the end-user. Therefore, OrthoGuide is positioned not as a replacement for dynamic analysis pipelines, but as a tool for rapid hypothesis generation and initial data exploration within its defined evolutionary context.

## Materials and Methods

### Database Implementation

The core of the OrthoGuide database consists of precomputed evolutionary rooting information for all annotated genes of eight eukaryotic model species (Table 1). This was carried out systematically applying the Bridge algorithm, implemented in the GeneBridge R package (Campos et al. 2024), to each gene’s corresponding OG.

**Table 1:** 8 representatives of the 360 species included in the OrthoGuide database v3

Species name	Number of genes	Number of COGs
*Homo sapiens*	19,452	7,702
*Mus musculus*	20,812	7,445
*Rattus norvegicus*	22,072	7,390
*Danio rerio*	25,294	7,040
*Drosophila melanogaster*	12,725	6,369
*Caenorhabditis elegans*	17,698	8,227
*Arabidopsis thaliana*	23,348	6,962
*Saccharomyces cerevisiae*	5,534	3414

The full table for all species can be found in Supplementary Table 2.

To ensure compatibility with external genomic resources, the analysis was performed against a eukaryotic phylogenetic tree that synchronizes three key data sources: the tree topology was derived from TimeTree (Kumar et al. 2017), augmented with species assignments from the NCBI Taxonomy, and filtered to include only eukaryotes present in STRING database v11.0 (Szklarczyk et al. 2021). We retrieved full taxonomic lineages for all species via the NCBI Entrez API. The “root” clades in the final database represent the latest common ancestor between each reference species and its relatives. These nodes were sequentially indexed by their evolutionary distance from the reference organism, and assigned informative names by extracting the most specific, nonredundant rank from their NCBI lineage strings.

The primary inputs for the rooting inference consisted of gene-to-COG mappings and a eukaryotic phylogenetic tree. We obtained the orthology data by mapping all genes available in the STRING database v11.0 (Szklarczyk et al. 2021) to their respective Clusters of Orthologous Groups (COG) identifiers using the official STRING mapping files. These mappings provided the phyletic patterns (the binary presence/absence matrix) across species required for the Bridge algorithm. The phyloTree object was constructed using TimeTree as a topological backbone, encompassing all eukaryotic species annotated within the reference dataset to maintain identifier consistency.

Parameters for the GeneBridge execution were set based on the algorithm’s prior benchmarks, utilizing a probability threshold (*h*) of 0.5 to guide the iterative root search and a penalty factor (*g*) of 2 to account for the higher relative frequency of gene loss over gene gain. Statistical significance for each root assignment was estimated using 1,000 permutations. To ensure reproducibility and facilitate future updates, the entire database generation workflow was encapsulated into a containerized Nextflow pipeline (Langer et al. 2025).

All organisms are organized into separate tables in the SQLite3 database, each table being named after the organism’s NCBI taxonomy identifier. All tables contain the same columns, describing the orthologous root inference (Table 2).

**Table 2:** OrthoGuide database schema, containing the columns present in each organism’s table and their data types

Column Name	Data Type	Description	Example
cog_id	TEXT	The Cluster of Orthologous Groups (COG) identifier.	NOG106405
root	INTEGER	The numerical ID representing the last common ancestor (LCA) or root clade.	1
clade_name	TEXT	The descriptive name of the root clade.	Homo
protein_id	TEXT	The STRING protein identifier for the specific protein.	ENSP00000490314
preferred_name	TEXT	The common gene name (e.g. HGNC symbol) used for querying.	BRCA1

### Web Application

The complete set of rooting results is stored in a SQLite3 database, which is accessed via a static, single-page application built with the Vue 3 JavaScript framework. The use of SQLite WebAssembly enables all database queries to be executed entirely on the client-side.

Interactive network visualizations are implemented using the D3.js library, which manages a force-directed simulation to represent protein-protein interactions. Interaction data, including confidence scores, are retrieved from the STRING database. When a user selects a specific ancestral node via the integrated slider, the application dynamically filters the network to display only the subset of proteins that had emerged by that point in evolutionary history. Nodes are color-coded to provide evolutionary context: proteins rooted exactly at the selected ancestral node are shown in orange, while those rooted in more ancient clades are shown in blue.

To ensure the visualization remains biologically informative even for sparsely connected systems, genes without interactions in the selected set are automatically grouped and displayed in a separate, draggable container. Furthermore, the application caches node coordinates after the initial simulation. This allows users to move between ancestral clades while maintaining a stable and consistent layout. All visualizations can be exported directly from the browser as either SVG or Portable Network Graphics (PNG) files.

## Supplementary Material

evag119_Supplementary_Data

## Data Availability

The OrthoGuide database is available at https://github.com/dalmolingroup/orthoguide. The reference tree used for computing the evolutionary information present in the database is available at https://doi.org/10.5281/zenodo.18758268.

## Software Availability

GeneBridge is released under the Artistic-2.0 license. The source code for GeneBridge is available at https://github.com/sysbiolab/GeneBridge. OrthoGuide is released under the Apache 2.0 license and its source code is available at https://github.com/dalmolingroup/orthoguide/. The OrthoGuide application can be found at https://dalmolingroup.imd.ufrn.br/orthoguide/.
